# POU1F1 transcription factor induces metabolic reprogramming and breast cancer progression via LDHA regulation

**DOI:** 10.1038/s41388-021-01740-6

**Published:** 2021-03-13

**Authors:** Anxo Martínez-Ordoñez, Samuel Seoane, Leandro Avila, Noemi Eiro, Manuel Macía, Efigenia Arias, Fabio Pereira, Tomas García-Caballero, Noemi Gómez-Lado, Pablo Aguiar, Francisco Vizoso, Román Perez-Fernandez

**Affiliations:** 1 Department of Physiology-Center for Research in Molecular Medicine and Chronic Diseases (CIMUS), University of Santiago de Compostela, Santiago de Compostela, Spain; 2 Research Unit, Hospital Fundación de Jove, Gijón, Spain; 3 Department of Obstetrics and Gynecology, Health Research Institute of Santiago de Compostela (IDIS)-University of Santiago de Compostela, Santiago de Compostela, Spain; 4 Department of Radiation Oncology, Health Research Institute of Santiago de Compostela (IDIS)-University of Santiago de Compostela, Santiago de Compostela, Spain; 5 Department of Morphological Sciences, Health Research Institute of Santiago de Compostela (IDIS)-University of Santiago de Compostela, Santiago de Compostela, Spain; 6 Molecular Imaging Group. Department of Psychiatry, Radiology, Public Health, Nursing and Medicine, and Health Research Institute of Santiago de Compostela (IDIS). University of Santiago de Compostela, Santiago de Compostela, Spain; 7 Present Address: Department of Pathology and Laboratory Medicine, Weill Cornell Medicine, New York, NY USA

**Keywords:** Breast cancer, Mechanisms of disease

## Abstract

Metabolic reprogramming is considered hallmarks of cancer. Aerobic glycolysis in tumors cells has been well-known for almost a century, but specific factors that regulate lactate generation and the effects of lactate in both cancer cells and stroma are not yet well understood. In the present study using breast cancer cell lines, human primary cultures of breast tumors, and immune deficient murine models, we demonstrate that the POU1F1 transcription factor is functionally and clinically related to both metabolic reprogramming in breast cancer cells and fibroblasts activation. Mechanistically, we demonstrate that POU1F1 transcriptionally regulates the lactate dehydrogenase A (LDHA) gene. LDHA catalyzes pyruvate into lactate instead of leading into the tricarboxylic acid cycle. Lactate increases breast cancer cell proliferation, migration, and invasion. In addition, it activates normal-associated fibroblasts (NAFs) into cancer-associated fibroblasts (CAFs). Conversely, LDHA knockdown in breast cancer cells that overexpress POU1F1 decreases tumor volume and [^18^F]FDG uptake in tumor xenografts of mice. Clinically, POU1F1 and LDHA expression correlate with relapse- and metastasis-free survival. Our data indicate that POU1F1 induces a metabolic reprogramming through LDHA regulation in human breast tumor cells, modifying the phenotype of both cancer cells and fibroblasts to promote cancer progression.

## Introduction

Cellular metabolism reprogramming is a hallmark of cancer cells [1, 2]. Metabolic plasticity includes aerobic glycolysis, also called the Warburg effect, a well-known feature of tumors early recognized by Otto Warburg [3]. The glycolysis occurring in cancer cells gives rise to a high-rate glucose uptake and the conversion of pyruvate into lactate, instead of converting into Acetyl CoA and leading to the tricarboxylic acid (TCA) cycle and mitochondrial oxidative phosphorylation (OXPHOS). Moreover, the lactate output to the extracellular space results in acidification of tumor microenvironment (TME) [4–6]. In addition to the key role of lactate in tumor cell maintenance [7, 8], lactate is known to affect stromal cells in the TME. For example, in immune cells, lactate induces M2-like polarization of tumor-associated macrophages (TAM) and TAM-induced angiogenesis [9]. It also impairs T and natural killer cell activation and compromises dendritic cell differentiation and maturation that suppresses anticancer immune responses [10, 11]. In endothelial cells, lactate has been demonstrated to increase angiogenesis [12]. However, the role of lactate on other stromal cell populations has not yet been fully elucidated. For example, administration of exogenous lactate in breast cancer cells has been shown to induce the cancer stem cell (CSC) phenotype [13], but it is not clear which source of lactate could contribute to inducing stemness: the cancer cell-derived lactate, the CSC-produced lactate, or both. Regarding fibroblasts, cancer cells reprogram the surrounding fibroblasts to induce aerobic glycolysis. In turn, fibroblasts produce lactate that can be taken up by cancer cells and oxidized in the mitochondria for energy production and tumor progression. This metabolic coupling is referred to as the “reverse Warburg effect” [14]. In addition, a recent study indicated that lactate secreted by pancreatic ductal adenocarcinoma cells reprogram mesenchymal stem cells epigenetically to differentiate into cancer-associated fibroblasts (CAFs), thus leading to tumor progression [15].

Lactate dehydrogenase A (LDHA) is the key enzyme generating lactate from pyruvate. HIF-1, c-Myc, and p53 are likely the most widely studied lactate regulators. Upregulation of HIF-1 and c-Myc and suppression of p53 are responsible for the metabolic switch to glycolysis in cancer cells [16]. LDHA overexpression promotes cell proliferation and invasion in pituitary adenomas [17], whereas reduction in LDHA activity compromises the ability of tumor cells to proliferate under hypoxia and severely diminishes tumorigenicity and tumor cell maintenance [7].

Transcriptional regulation of prolactin (PRL) and growth hormone (GH) in the pituitary gland by the POU1F1 transcription factor is well known [18, 19]. However, POU1F1 is also expressed in non-pituitary tissues, such as human breast, where it also regulates GH and PRL [20, 21]. High POU1F1 levels in breast cancer cells induce cell proliferation, reduce apoptosis, and increase migration and invasion [22]. In fact, high expression of POU1F1 in breast cancer correlates with poor clinical outcome [23]. Hypomethylation profiles in clusters of circulating breast cancer cells have recently been identified in binding sites for several transcription factors related to stemness and proliferation, including POU1F1. This epigenetic mechanism has been related to metastasis seeding [24].

The current study analyzes the role of POU1F1 in breast cancer cell metabolism. Human breast cancer cell lines and primary human breast tumors were used to evaluate the effect of POU1F1 overexpression and POU1F1 knockdown in the glycolysis pathway. Using immunodeficient mice, we studied how cancer cells with POU1F1 overexpression and LDHA blockade could affect tumor growth and glucose uptake. In patients, POU1F1 and LDHA mRNA expression was correlated with breast cancer clinical outcome. Finally, in primary cultures of human breast tumors, we studied the effect of both POU1F1 and LDHA on fibroblast activation.

## Results

### POU1F1 in breast cancer cells induces metabolic reprogramming

**Fig. 1:**
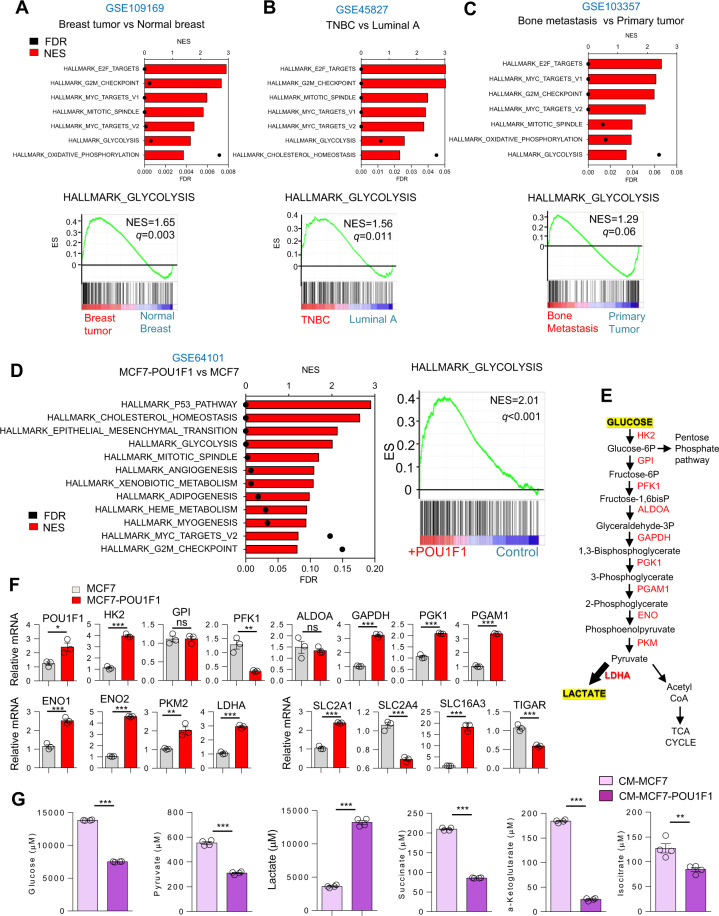
POU1F1 induces metabolic reprogramming in human breast adenocarcinoma cells. **A**–**C** Dataset enrichment graphs related with metabolic and proliferation process categories from Hallmark collection (up) and GSEA plot of enrichment in Glycolysis geneset (down). Gene expression data obtained from GSE109169, GSE45827, and GSE103357 as indicated: **A** Tumor breast cancer patient samples (*n* = 25) vs normal breast (*n* = 25); **B** TNBC breast tumors (*n* = 41) vs Luminal A breast tumors (*n* = 29); **C** Breast cell line derived from bone metastasis patient (*n* = 3) vs primary tumor cells (*n* = 2). FDR false discovery rate, NES normalized enrichment score. **D** Dataset enrichment graphs related with metabolic, proliferation, and development process categories from Hallmark collection (left) and GSEA plot of enrichment in Glycolysis geneset (right) of microarray data from POU1F1-overexpressing MCF7 cells vs MCF7 cells (GSE64101). **E** Glycolysis pathway and catalytic enzymes (in red). **F** qPCR of glycolytic enzymes, carriers, and factors involved in the glycolytic pathway in control and POU1F1-overexpressing MCF7 cells. *POU1F1*, *HK2* (hexokinase 2), *GPI* (glucose-6-phosphate isomerase), *PFK1* (6-phosphofructokinase), *ALDOA* (aldolase A), *GAPDH* (glyceraldehyde 3-phosphate dehydrogenase), PGK1 (phosphoglycerate kinase 1), *PGAM1* (phosphoglycerate mutase), *ENO1* (enolase 1), *ENO2*, *PKM2* (pyruvate kinase M2), *LDHA* (lactate dehydrogenase A), *SLC2A1* (glucose transporter 1, GLUT1), *SLC2A4* (GLUT4), *SLC16A3* (monocarboxylate transporter 4, MCT4), and *C12orf5* (TIGAR). **G** ^1^H-NMR assay of glycolytic- and TCA-derived metabolites in conditioned medium (CM) of MCF7 and MCF7-POU1F1 cells after 24 h culture. *FDR-adjusted *p* value (*q*-value) < 0.05; **< 0.01; ***0.001; ns not significant.

**Fig. 2:**
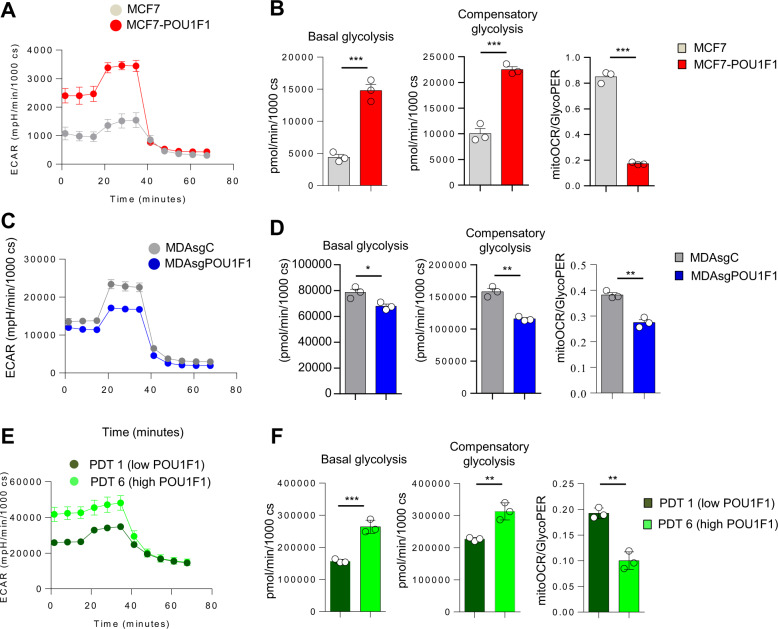
POU1F1 regulates the glycolytic profile of breast cancer cell lines and primary tumors in vitro. **A** Representative ECAR Glycolytic Rate Assay profile in MCF7 and MCF7-POU1F1 cells. cs cells. **B** Quantification of basal glycolysis, compensatory glycolysis and mitoOCR/glycoPER basal ratio in MCF7 and MCF7-POU1F1 cells. **C** Representative ECAR Glycolytic Rate Assay profile in control (MDAsgC) and POU1F1-knocked-out MDA-MB-231 (MDAsgPOU1F1) cells. **D** Quantification of basal glycolysis, compensatory glycolysis and mitoOCR/glycoPER basal ratio in MDAsgC and MDAsgPOU1F1 cells. **E** Representative ECAR Glycolytic Rate Assay profile in human primary breast tumor-derived cultures (see Fig. 6D) with low POU1F1 expression (PDT 1) and high POU1F1 expression (PDT 6). **F** Quantification of basal glycolysis, compensatory glycolysis and mitoOCR/glycoPER basal ratio in PDT 1 and PDT 6. Data are expressed as mean ± SEM. *P < 0.05, **P < 0.01, and ***P < 0.001.

Bioinformatic analyses of human breast cancer datasets were carried out for glycolytic activity and other processes related with proliferation and metabolism (Fig. 1A–C). Glycolytic activity was found to be higher in breast tumors than in normal breast (GSE109169), in triple-negative breast cancer (TNBC) than in luminal A tumors (GSE45827), and in bone metastasis than in primary tumors (GSE103357). Interestingly, glycolytic activity was also found to be higher in the breast cancer subtypes Luminal B and HER2 as compared to luminal A (Supplementary Fig. S1A–B). Given that POU1F1 is related to pituitary gland development and cancer progression, we analyzed these biological processes in the context of breast cancer. We carried out an unbiased transcriptomic analysis by performing a microarray (GSE64101) in the control luminal A subtype of MCF7 cells with low endogenous POU1F1 expression (MCF7) and after transient transfection by a POU1F1 overexpression vector (MCF7-POU1F1). To analyze these data, a gene-set enrichment analyses was performed. We found that POU1F1 overexpression induced a clear enrichment of glycolysis signature (NES = 2.01, *P* < 0.001) (Fig. 1D). Next, we selected well-known enzymes and carriers involved in the glycolytic pathway (Fig. 1E) and a real-time PCR was carried out before and after POU1F1 overexpression. Our data confirmed significant upregulation in mRNA expression of eight glycolysis-related genes: *HK2* (hexokinase 2), *GAPDH* (glyceraldehyde 3-phosphate dehydrogenase), *PGK1* (phosphoglycerate kinase 1), *PGAM1* (phosphoglycerate mutase), *ENO1* (enolase 1), *ENO2*, *PKM2* (pyruvate kinase M2), and *LDHA* (lactate dehydrogenase A). Downregulation of *PFK1* (6-phosphofructokinase) and no changes of *GPI* (glucose-6-phosphate isomerase), or *ALDOA* (aldolase A) glycolysis-related genes (Fig. 1F) were observed. In addition, two key transporters in glucose metabolism, *SLC2A1* (glucose transporter 1, GLUT1), and *SLC2A4* (GLUT4*)*, significantly decreased and increased, respectively, after POU1F1 overexpression. The mRNA expression of *SLC16A3* (monocarboxylate transporter 4, MCT4), which is responsible for transporting lactate out of cells, also significantly increased after POU1F1 overexpression (Fig. 1F). Furthermore, the *C12orf5* gene which codes for the TP53-inducible glycolysis and apoptosis regulator (TIGAR) protein, a well-known glycolysis p53-mediator [25, 26], decreases after POU1F1 overexpression, and this could suggest a possible role for TIGAR in POU1F1-induced glycolysis. To analyze possible changes in secreted metabolites, conditioned medium (CM) from MCF7 and MCF7-POU1F1 cells after 24 h of culture was analyzed by ^1^H-NMR assay (Supplementary Fig. S2, A-B, and Supplementary Table S1). Glycolytic- and TCA-derived metabolites showed dramatic changes after POU1F1 overexpression, with a significant decrease in glucose and a significant increase in lactate (both *P* < 0.001) (Fig. 1G). Several TCA-derived metabolites also presented significantly reduced levels in CM-MCF7-POU1F1 cells (Fig. 1G). Altogether, our data indicate a clear Warburg effect induced by POU1F1., i.e., pyruvate to lactate production increased, while pyruvate to TCA-cycle products decreased at least at the extracellular level. To confirm the functional relevance of our findings, glycolytic activity of breast cancer cells was assayed by measuring extracellular acidification rate (ECAR), quantification of basal glycolysis, compensatory glycolysis, and mitochondrial oxygen consumption rate (OCR)/proton extrusion rate (glycoPER) basal ratio in both MCF7 and MCF7-POU1F1 cells. The data obtained show that POU1F1 significantly increased the glycolytic profile as compared to control MCF7 cells (Fig. 2A, B). Conversely, knockout of POU1F1 (MDAsgPOU1F1) using the CRISPR methodology in the MDA-MB-231 cell line (triple-negative subtype, with high endogenous levels of POU1F1) presented a significant decrease in the glycolytic profile compared to control cells (MDAsgC) (Fig. 2C, D and Supplementary Fig. S3). Primary cultures of two human breast tumors with low POU1F1 protein expression (PDT 1) and high expression (PDT 6) (see below) were selected for glycolytic activity analyses. As shown in Fig. 2E, F, similar data were obtained for the human breast tumors and the breast cancer cell lines, which strongly suggests that POU1F1 regulates the glycolysis pathway in breast tumors.

### POU1F1 regulates LDHA expression in breast cancer

**Fig. 3:**
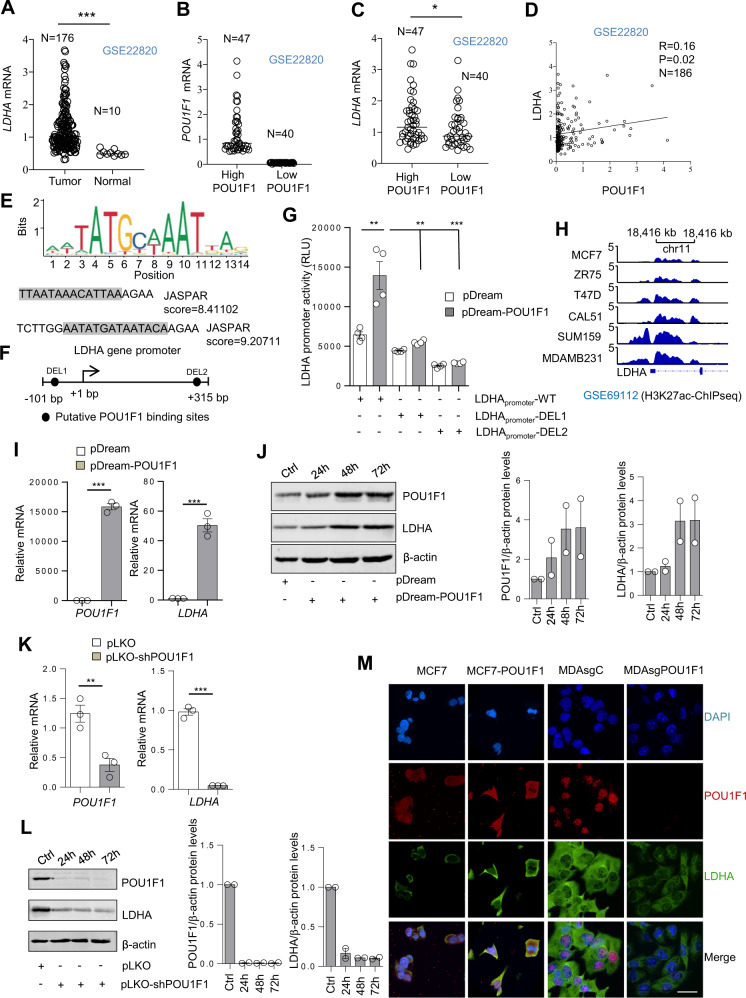
POU1F1 regulates LDHA expression. **A** Dispersion plot of LDHA mRNA levels in human breast tumors (*n* = 176) and normal tissues (*n* = 10) (GSE22820). **B**, **C** *POU1F1* and *LDHA* mRNA levels according to *POU1F1* mRNA levels (high POU1F1: higher than 75th percentile, and lower POU1F1: with levels below 25th percentile). **D** Spearman correlation analysis of *POU1F1* and *LDHA* mRNA expression (log2) (*n* = 186). **E**, **F** JASPAR analysis indicates two POU1F1 binding sites in the LDHA gene at positions −76/−62 bp and +276/+290 bp with respect to transcription start site. DEL1 and DEL2 indicate deletion of POU1F1 binding sites. **G** The wild LDHA promoter (−101 to +315 bp from the transcription start site; LDHA_promoter_-WT) and the LDHA promoter with specific deletions at the POU1F1 binding sites (LDHA_promoter_-DEL1 and LDHA_promoter_-DEL2) were subcloned into the pRP vector, transfected in HEK 293 cells, and co-transfected with the control (pDream) or the pDream-POU1F1 overexpression vector for 48 h, and luciferase activity was measured. Normalized relative luciferase units (RLU) were calculated as the ratio of luciferase activity in control and POU1F1-transfected cells. **H** H3K27Ac occupancy of LDHA promoter in human breast cancer cell lines (GSE69112), analyzed by ChIPseq (MCF7, ZR75 and T47D: Luminal A; CAL51, SUM159, and MDA-MB-231: Triple Negative Breast Cancer). **I** MCF7 cells were transiently transfected with the pDream-POU1F1 overexpression vector and 72 h later a qPCR was carried out to evaluate *LDHA* and *POU1F1* mRNA expression. **J** Western blot of LDHA, POU1F1, and β-actin in MCF7 cells after transient POU1F1 overexpression (*n* = 2). **K** *LDHA* mRNA in MDA-MB-231 cells after POU1F1 knockdown for 72 h. **L** WB of POU1F1, LDHA, and β-actin in MDA-MB-231 cells after POU1F1 knockdown (*n* = 2). **M** Representative images of POU1F1 and LDHA after confocal microscopy in MCF7 and MDA-MB-231 cells after stable POU1F1 overexpression (MCF7-POU1F1) and POU1F1 knockout (MDAsgPOU1F1). DAPI was used as nuclei marker. Scale bar: 15 μm. Data are expressed as mean ± SEM. **P < 0.01, and ***P < 0.001.

LDHA is a key enzyme in the glycolysis pathway, transforming pyruvate into lactate. Aberrantly high expression of LDHA has been demonstrated in multiple cancers, including breast cancer, and it has been associated with malignant tumor progression. In fact, bioinformatic search of human breast cancer tumors and normal breast samples (GSE22820) revealed a significant (*P* < 0.001) increase of *LDHA* mRNA expression in human breast tumors as compared with normal mammary tissue (Fig. 3A). Using the same database, we also classified breast tumors and normal samples according to *POU1F1* mRNA expression (Fig. 3B). Further analyses also showed a significant (*P* < 0.05) correlation between *POU1F1* and *LDHA* mRNA expression (Fig. 3C, D).

In order to evaluate the hypothesis that POU1F1 could regulate the LDHA gene at transcriptional level, we searched for potential binding sites of POU1F1 in the *LDHA* promoter using the JASPAR database (http://jaspar.genereg.net/), and found two putative motifs for POU1F1 in the *LDHA* gene promoter region (Fig. 3E). These two consensus elements for POU1F1 were located at −76 to −62 bp upstream and +276 to +290 bp downstream from the start transcription site in the *LDHA* promoter (Fig. 3F). To evaluate the possible transcriptional regulation of *LDHA* by POU1F1, a luciferase reporter assay in the absence or presence of POU1F1 was carried out using three plasmids: (a) the wild *LDHA* promoter (LDHA_promoter_-WT, −101 to +315 bp), (b) a plasmid with deletion of the first POU1F1 binding site (LDHA_promoter_-DEL1, deletion of −76 to −62 bp), and (c) a plasmid with deletion of the second POU1F1 binding site (LDHA_promoter_-DEL2, deletion of +276 to +290 bp). HEK 293 cells co-transfected with the LDHA_promoter_-WT and the POU1F1 overexpression vector significantly increased luciferase activity as compared with control (*P* < 0.01), whereas deletion of both the first and the second POU1F1 binding sites significantly reduced luciferase activity (*P* < 0.01 and *P* < 0.001) (Fig. 3G). In fact, interrogation of the epigenetic status of LDHA gene promoter in breast cancer cell lines showed a peak enrichment in H3K27ac, an epigenetic mark associated with higher activation of transcription (Fig. 3H). Furthermore, transient POU1F1 overexpression in MCF7 cells significantly (*P* < 0.001) increased *LDHA* mRNA expression (Fig. 3I). Western blots also indicated a visible increase in LDHA protein expression after POU1F1 overexpression in both transient (Fig. 3J) and stable (Supplementary Fig. S4A) MCF7 cells. Conversely, transient knockdown of POU1F1 using a pLKO-shPOU1F1 vector in MDA-MB-231 cells significantly decreased *LDHA* mRNA (*P* < 0.001, Fig. 3K) and protein (Fig. 3L) levels. Confocal microscopy of MCF7 control cells transfected stably with the pTRE2 control vector (MCF7), MCF7 cells transfected stably with the pTRE2-POU1F1 vector (MCF7-POU1F1), MDA-MB-231 control cells transfected with the pLentiCRISPRv2 control (MDAsgC), and after POU1F1 knockout (MDAsgPOU1F1), indicated a correlation between POU1F1 (in red) and LDHA (in green) immunodetection (Fig. 3M). Supplementary Fig. S4B also shows a decrease in LDHA protein expression after knockout of POU1F1 in MDA-MB-231 cells using three different CRISPR clones.

### POU1F1-induced lactate facilitates cancer progression in breast cancer cells

**Fig. 4:**
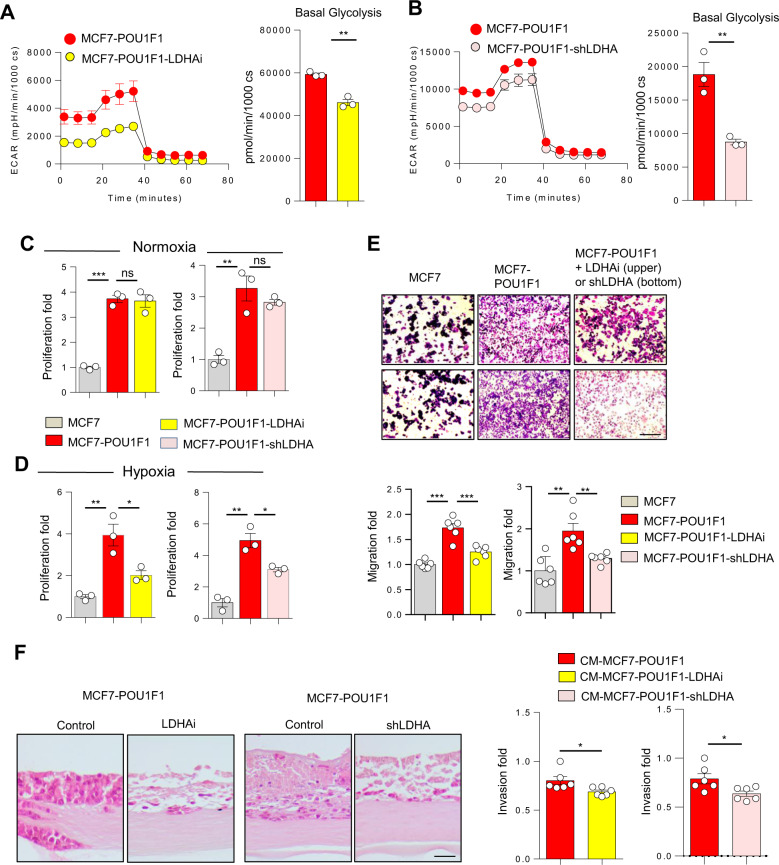
POU1F1-induced lactate acts on cancer cells to induce cancer progression. **A**, **B** Administration of a LDHA inhibitor (10 μM, GSK2837808A, referred as LDHAi) or LDHA knockdown (shLDHA) in MCF7-POU1F1 cells for 24 h significantly decreases extracellular acidification rate (ECAR) and basal glycolysis. Data were obtained using an XFp seahorse cell analyzer. **C**, **D** Cell proliferation in control and POU1F1-overexpressing MCF7 cells treated with 10 μM LDHAi or transfected with the pKLO-shLDHA vector for 72 h under normoxic and hypoxic (1% O_2_) atmosphere. **E** Representative figure and quantitative analysis of trans-well migration assays in control cells (MCF7), POU1F1-overexpressing cells (MCF7-POU1F1), and MCF7-POU1F1 cells either treated with LDHAi (upper panel) or after LDHA knockdown (shLDHA, bottom panel). Scale bar: 100 μm. **F** Representative images of H&E-stained sections of MCF7-POU1F1 cells cultured in an organotypic system in the presence of a pharmacological LDHA inhibitor (LDHAi) or LDHA knockdown (shLDHA), and quantification of cell invasion. Data are expressed as mean ± SEM, *P < 0.05, **P < 0.01, ***P < 0.001. Scale bar: 50 μm.

To evaluate the effect of lactate on breast cancer cells, glycolytic activity (ECAR and basal glycolysis) was assayed in stable MCF7 overexpressing POU1F1 cells before and after treatment with either a pharmacological LDHA inhibitor (GSK2837808A, referred as LDHAi, 10 μM for 24 h) or after genetic LDHA knockdown (shLDHA, Supplementary Fig. S4C). Figure 4A, B indicate a significant decrease in ECAR and basal glycolysis in MCF7-POU1F1 cells after both LDHA procedures. Previous studies have shown an LDHA-dependence on cancer cell proliferation under hypoxic environment conditions [27]. Our data are in line with these reports. Proliferation of MCF7 cells significantly increased after POU1F1 overexpression in both normoxic and hypoxic cultures, as we previously demonstrated [28], but only under hypoxia does the treatment of MCF7-POU1F1 cells with the LDHAi and knockdown of LDHA significantly (*P* < 0.05) reduce cell proliferation (Fig. 4C, D). Next, migration of MCF7-POU1F1 cells was evaluated using a trans-well assay. A significant increase in cell migration was observed in MCF7 cells after POU1F1 overexpression as compared with control cells (Fig. 4E). However, treatment either with LDHAi (*P* < 0.001) or shLDHA (*P* < 0.01) significantly reduced cell migration (Fig. 4E). Finally, to analyze cancer cell invasion, we carried out organotypic cultures using control MCF7-POU1F1 cells and MCF7-POU1F1 cells either treated with LDHAi or transfected with shLDHA. Our data indicate a significant decrease (*P* < 0.05) in cell invasion after both experimental manipulations (Fig. 4F). In summary, our data suggest that either blocking LDHA enzymatic activity or decreasing LDHA expression in MCF7-POU1F1 cells reduces proliferation, migration, and invasion of breast cancer cells.

### POU1F1-regulated LDHA influences tumor growth and tumor glucose uptake in vivo, and both POU1F1 and LDHA expression are related to clinical outcome

**Fig. 5:**
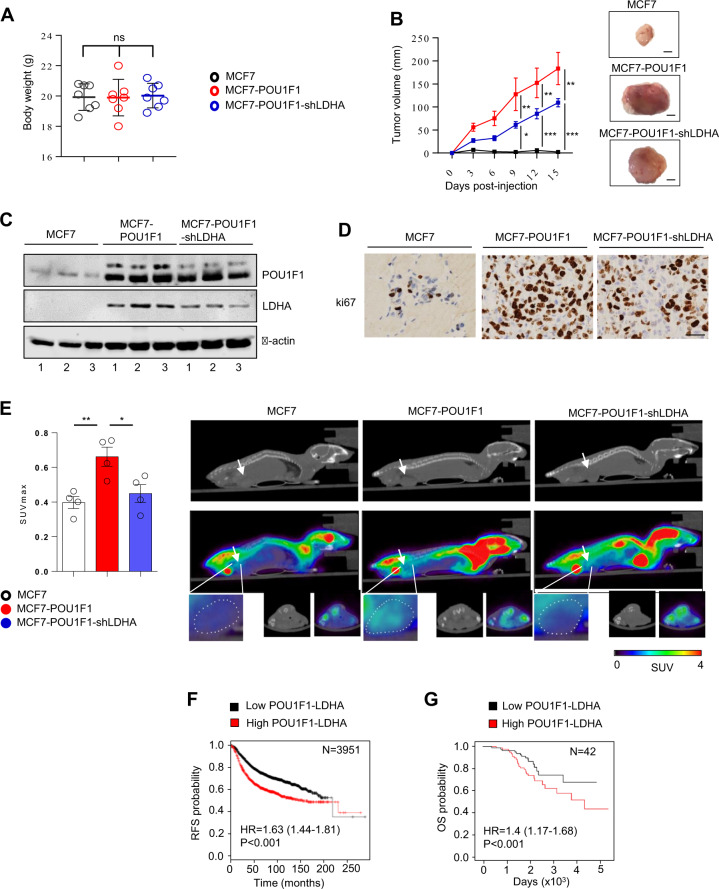
LDHA knockdown in POU1F-overexpressing cancer cells reduces tumor growth, and tumor glucose uptake in vivo. **A** Body weight of mice orthotopically injected with MCF7, MCF7-POU1F1, and MCF7-POU1F1-shLDHA human breast cancer cells (*n* = 7 mice per group). **B** Tumor volume of mice injected with MCF7, MCF7-POU1F1, and MCF7-POU1F1-shLDHA cells and representative images of tumors. Scale bar: 1000 μm. **C** qWB analysis of POU1F1, LDHA and β-actin in tumors from nine mice injected with MCF7 (*n* = 3), MCF7-POU1F1 (*n* = 3) and MCF7-POU1F1-shLDHA cells (*n* = 3). **D** ki67 immunostaining of mouse tumors. Scale bar: 50 μm. **E** On the left, SUV_max_ values for the three experimental groups (*n* = 4 mice per group). Significant differences can be observed between MCF7-POU1F1 and other groups (*P < 0.05, **P < 0.01). On the right, [^18^F]FDG PET/CT images from representative BALB/c-nu mice. Metabolic activity is coded on a color scale ranging from blue (low [^18^F]FDG uptake) to red (high [^18^F]FDG uptake). At the top, xenograft tumors indicated by arrows on CT images. At the bottom, tumors delineated using PET/CT images. Axial views are also shown. **F** Correlation between *POU1F1* and *LDHA* mRNA expression and relapse-free survival (RFS) in human breast tumors (*n* = 3951). **G** Correlation between *POU1F1* and *LDHA* mRNA expression and overall survival (OS) in human breast tumors (*n* = 42). Analysis of (**F**) and (**G**) were done using the KM plotter and the ProgGeneV2 online tools, respectively.

To evaluate in vivo the effect of LDHA knockdown in POU1F1-overexpressing cells, immunodeficient BALB/c-nu mice were injected with control MCF7 cells (stably transfected with the pTRE2 control vector, *n* = 7, MCF7), MCF7 cells with POU1F1 overexpression (pTRE2-POU1F1-overexpressing vector plus the pLKO control vector, *n* = 7, MCF7-POU1F1), and with MCF7 cells with POU1F1 overexpression and LDHA knockdown (pTRE2-POU1F1-overexpressing vector plus the pLKO-shLDHA plasmid, *n* = 7, MCF7-POU1F1-shLDHA). Xenografted tumors were monitored every 3 days, and mice were sacrificed at day 15 post injection. Body weight remained stable among all three groups (Fig. 5A). Tumor volume in MCF7-injected mice was negligible at day 15, but a continuous growth of tumor volume in both the MCF7-POU1F1 and the MCF7-POU1F1-shLDHA groups was observed during the study, being significatively higher at 9, 12, and 15 days in MCF7-POU1F1 mice comparing to LDHA knocked-down mice (Fig. 5B). POU1F1 and LDHA protein levels in three tumors from each group were assayed by western blot showing, as expected, reduced levels of LDHA in MCF7-POU1F1-shLDHA-injected mice with respect to MCF7-POU1F1 group (Fig. 5C). Immunohistochemistry analyses of tumor xenografts showed high cell proliferation (ki67 marker) in both MCF7-POU1F1 and MCF7-POU1F1-shLDHA groups with respect to MCF7-injected group, but ki67 staining was reduced in MCF7-POU1F1-shLDHA as compared to the MCF7-POU1F1 injected mice (Fig. 5D).

In addition, four mice per group were assayed for glucose uptake just before being sacrificed, using [^18^F]FDG PET/CT scans. SUV_max_ indicates a significant (*P* < 0.01) increase in glucose uptake in tumors from MCF7-POU1F1 injected mice as compared to control group (Fig. 5E). However, tumors in mice injected with MCF7-POU1F1-shLDHA cells had significantly reduced SUV_max_ (*P* < 0.05) as compared with MCF7-POU1F1 injected mice, but similar values to those found in control mice (Fig. 5E). Finally, to study the possible relationship between *POU1F1/LDHA* expression and clinical outcome, *POU1F1* and *LDHA* mRNA was analyzed in a dataset of human breast cancer patients. We found a significant correlation between *POU1F1/LDHA* expression and both relapse-free survival (RFS) (*P* < 0.001) and overall survival (OS) (*P* < 0.001) (Fig. 5F, G, and Supplementary Fig. S5).

### POU1F1 expression in breast cancer tumor samples correlates with CAF activation

**Fig. 6:**
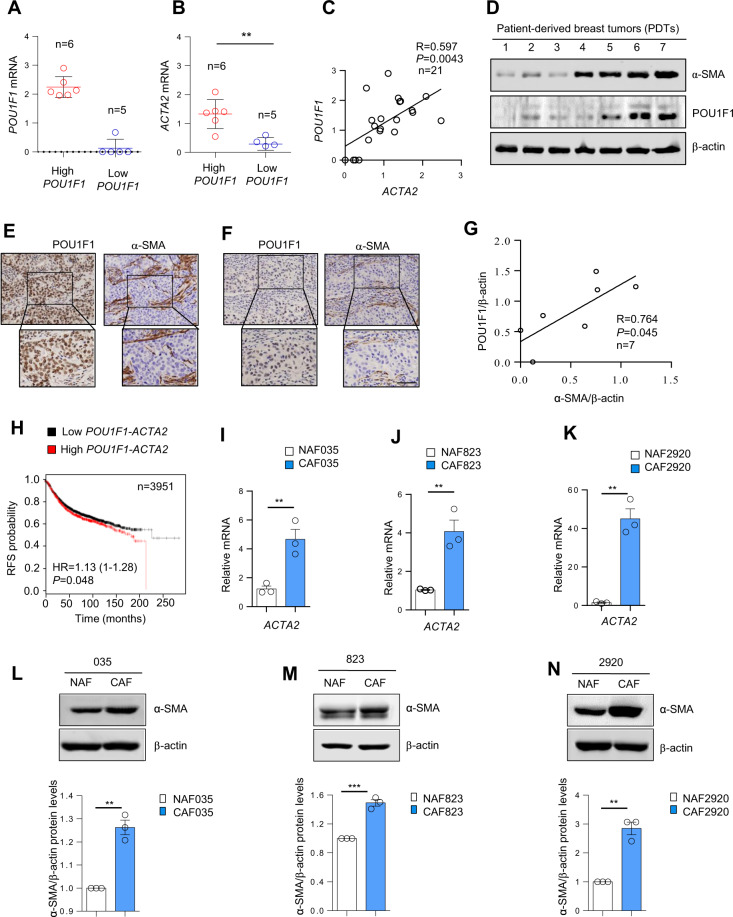
POU1F1 expression correlates with stromal activation in breast cancer tumor samples. **A**–**C** *POU1F1* and *ACTA2* mRNA expression in 21 breast tumor samples were evaluated by qPCR and plotted by POU1F1 expression (high POU1F1: higher than 75th percentile, low POU1F1: below 25th percentile). Dispersion plot indicates a significant positive correlation between *POU1F1* and *ACTA2* mRNA. **P < 0.01. **D** Quantitative western blot (qWB) of POU1F1, α-SMA, and β-actin (as loading control) in seven patient-derived breast tumors (PDT 1–7). **E** Representative immunostaining of POU1F1 in tumor cells and α-SMA in fibroblasts in human breast invasive ductal carcinomas with high POU1F1 expression. Scale bar: 75 μm. **F** Representative immunostaining of POU1F1 in tumor cells and α-SMA in fibroblasts in human breast invasive ductal carcinomas with low POU1F1 expression. Scale bar: 75 μm. **G** Dispersion plot indicates a significant correlation between POU1F1/β-actin and α-SMA /β-actin protein levels in seven patient-derived breast tumors. **H** *POU1F1* and *ACTA2* mRNA expression significantly correlate with relapse-free survival (RFS) in human breast tumor samples (*n* = 3951) (http://kmplot.com). **I**–**N** Characterization of normal-associated fibroblasts (NAFs, in the breast tumor surrounding tissue) and cancer-associated fibroblasts (CAFs, in the breast tumor tissue) in three human breast tumor samples based on *ACTA2* mRNA and α-SMA protein expression measured by qPCR and qWB. Data are expressed as mean ± SEM from three independent Western blots. **P < 0.01, ***P < 0.001.

Once we demonstrated that POU1F1 regulates LDHA and that pharmacological or genetic manipulation of LDHA induces phenotypic changes in breast cancer cells that modify cancer progression, we studied whether POU1F1 could have an impact on TME, specifically on fibroblasts. Numerous studies have shown increased NAF to CAF activation in breast cancer [29]. To study the role of POU1F1 in CAF activation, we first evaluated *POU1F1* mRNA expression in 21 human breast tumors (Supplementary Table S2). Tumor samples were classified as high POU1F1 (POU1F1 higher than 75th percentile, *n* = 6) and low POU1F1 (with levels below 25th percentile, *n* = 5) mRNA expression (Fig. 6A). Based on this classification, actin alpha 2 smooth muscle mRNA (*ACTA2)* expression was evaluated as a key marker of CAFs. We found a significant (*R* = 0.597, *P* = 0.0043) correlation between *POU1F1* and *ACTA2* mRNA (Fig. 6B, C). POU1F1 and ACTA2 protein (α-SMA) expression were also evaluated in seven primary cultures of human breast tumors (Supplementary Table S3). Both western blot and immunohistochemistry (IHC) showed a positive relationship between POU1F1 and α-SMA protein expression (Fig. 6D–F). Immunohistochemistry shows a representative example of POU1F1 and α-SMA immunodetection in two of the above-mentioned human breast tumors with high (Fig. 6E) and low (Fig. 6F) POU1F1 protein levels. As noted, POU1F1 is expressed at nuclear level in cancer cells, whereas α-SMA immunoreactivity in CAFs is present at cytoplasmic level. In line with the results at the mRNA level, a significant (*R* = 0.764, *P* = 0.045) correlation between POU1F1 and α-SMA protein expression was found (Fig. 6G). Indeed, a significant (*P* = 0.048, HR = 1.13 (1–1.28)) relationship between *POU1F1/ACTA2* mRNA levels and RFS was found in a human breast cancer dataset (*n* = 3951 samples) (Fig. 6H), which suggests a clinical prognostic value for both POU1F1 and α-SMA in breast tumors.

**Fig. 7:**
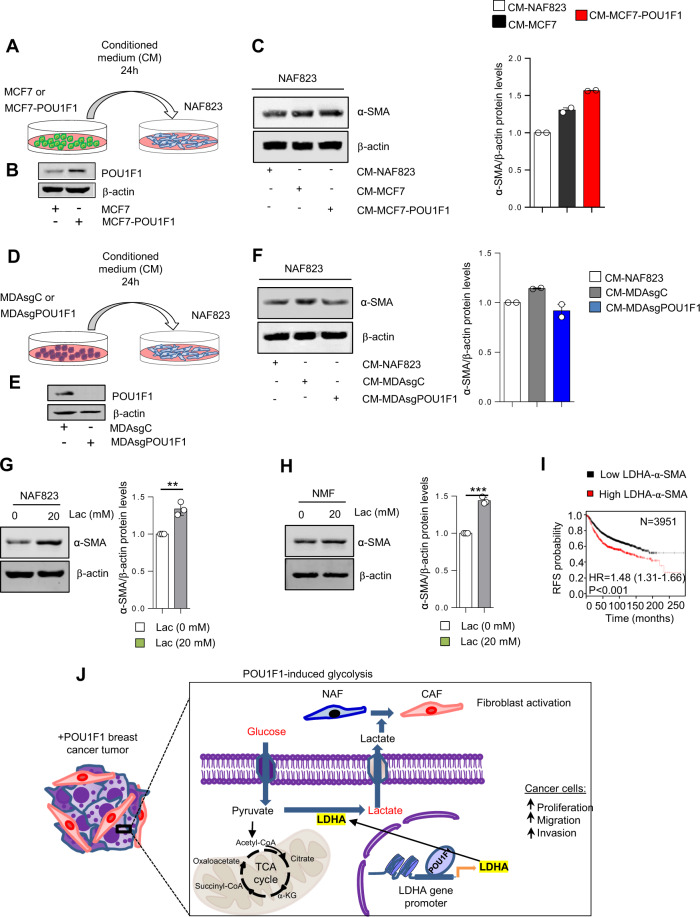
Lactate activates CAFs. **A** Conditioned medium (CM) from 24 h culture of NAF823, MCF7, and MCF7-POU1F1 cells was added to NAF823 for 24 h and α-SMA and β-actin protein levels were evaluated by qWB. **B** qWB of POU1F1 and β-actin in MCF7 and MCF7-POU1F1 cells. **C** qWB of α-SMA and β-actin in NAF823 after administration of CM-NAF823, CM-MCF7 and CM-MCF7-POU1F1 for 24 h, and representative histograms from two independent qWB. **D** CM from 24 h culture of NAF823, control MDA-MB-231 (MDAsgC), and MDA-MB-231 cells after POU1F1 knockout (MDAsgPOU1F1) was added to NAF823 for 24 h and α-SMA and β-actin were evaluated by qWB. **E** qWB of POU1F1 and β-actin in MDAsgC and MDAsgPOU1F1 cells. **F** qWB of α-SMA and β-actin in NAF823 after CM-NAF823, CM-MDAsgC, and CM-MDAsgPOU1F1 treatments, and representative histograms from two independent qWB. **G**–**H** Western blot of α-SMA and β-actin in NAF823 and normal mammary fibroblasts (NMFs) treated with vehicle (0) and lactate (Lac, 20 mM) for 24 h. Data are expressed as mean ± SEM from three independent quantitative western blots. **P < 0.01, and ***P < 0.001. **I** *LDHA* and *ACTA2* mRNA expression significantly correlate with relapse-free survival (RFS) in human breast tumor samples (http://kmplot.com). **J** Model of POU1F1-induced metabolic reprogramming and cancer progression. POU1F1 transcriptionally regulates LDHA leading pyruvate into lactate, which in turn increases proliferation, migration, and invasion of breast cancer cells and induces fibroblast activation.

To further evaluate the role of POU1F1 in NAF to CAF activation, we selected three human primary breast tumors: 035, 823, and 2920 (Supplementary Table S4). NAFs were obtained at least 2 cm away from the tumor. After tissue culture, both NAFs and CAFs were isolated and carefully characterized. Consistent with previous reports, fibroblasts were classified according to *ACTA2*/α-SMA mRNA/protein expression (NAFs have low *ACTA2*/α-SMA, and CAFs have high *ACTA2*/α-SMA) (Fig. 6I–N). In addition, other NAFs/CAFs markers, such as vimentin (VIM), fibroblast activation protein (FAP), vascular endothelial growth factor (VEGF), and C-X-C motif chemokine 12 (CXCL12) were evaluated, showing a significant increase in VIM, FAP, VEGF, and CXCL12 mRNA in CAFs with respect to NAFs (Supplementary Fig. S6A–C). MCF7 cells with and without POU1F1 overexpression were cultured for 24 h and CM from either MCF7 or MCF7-POU1F1 cultures was added to NAFs obtained from patient 823 (NAF823) to evaluate a possible NAF to CAF activation (Fig. 7A). Overexpression of POU1F1 (Fig. 7B) increased α-SMA protein levels in NAF823 (Fig. 7C), and conversely, treatment of NAFs with CM of MDA-MB-231 cells after knockout of POU1F1 (Fig. 7D, E) decreased α-SMA protein expression with respect to NAFs treated with CM-MDA-MB-231 control cells (Fig. 7F). Two similar experiments—treatment with CM from MCF7 cells before and after POU1F1 overexpression, and treatment with CM from MDA-MB-231 cells before and after POU1F1 knockout—were carried out in normal mammary fibroblasts (NMF) obtained from a normal patient after mammoplasty, confirming the results obtained with NAF823 (Supplementary Fig. S7A, B).

### POU1F1-induced lactate mediates CAF activation

To evaluate the effect of lactate on NAF to CAF activation, NAF823 and NMF were treated for 24 h with 20 mM lactate, and α-SMA protein levels were evaluated by western blot (Supplementary Fig. S8A). Both NAF823 and NMF showed a significant increase in α-SMA after lactate treatment with respect to untreated control fibroblasts (Fig. 7G, H). In fact, to correlate our in vitro data with clinical outcome, *LDHA*/*α-SMA* mRNA levels were analyzed in a breast cancer patient database (*n* = 3951) (http://kmplot.com). A significant (*P* < 0.001) correlation was found between *LDHA/*α*-SMA* and RFS (Fig. 7I). To analyze the effect of POU1F1 and lactate at protein level on CAF activation, CM was collected from a 24-h culture of stable MCF7-POU1F1 cells (control) treated either with 10 μM of an LDHA inhibitor (MCF7-POU1F1-LDHAi) or after LDHA knockdown (MCF7-POU1F1-shLDHA, see Supplementary Fig. S4C). Then, NAF823 and NMF were treated for 24 h with CM (Supplementary Fig. S8B, C), and α-SMA was immunoblotted to evaluate protein expression. The results were similar to those obtained after treatment with 20 mM lactate (Supplementary Fig. S8D–G).

## Discussion

The current study demonstrates that the POU1F1 transcription factor induces metabolic reprogramming by enhancing aerobic glycolysis of human breast cancer cells through transcriptional regulation of the *LDHA* gene (Fig. 7J). Elevated expression of LDHA increases conversion of pyruvate to lactate. In turn, the LDHA-dependent lactate increases proliferation of hypoxic cancer cells as well as migration and invasion. In addition, cancer cell-secreted lactate modifies fibroblast phenotype, which activates the conversion of NAF into CAF. LDHA downregulation in tumors of mice with POU1F1 overexpression significantly reduces tumor growth and tumor [^18^F]FDG uptake. In summary, our data revealed POU1F1 as a new factor of glycolysis regulation and demonstrate an important role for lactate in both cancer cells and fibroblasts to mediate cancer progression. Both POU1F1/LDHA and POU1F1/α-SMA expression correlate with clinical outcome, suggesting POU1F1 as a prognostic factor in breast cancer.

High POU1F1 expression in breast cancer cells is related to high cell proliferation, migration, invasion, and low apoptotic rate [22]. These features together with induction of EMT indicate that POU1F1 induces breast cancer progression [22]. In the present study, our first objective was to evaluate possible metabolic changes in breast cancer mediated by POU1F1. Increased glucose uptake relative to the surrounding normal tissues has emerged as a hallmark of cancer metabolism [30]. High glucose metabolism is accompanied by increased aerobic glycolysis in breast cancer cells, which produce lactate from pyruvate instead of transforming pyruvate into Acetyl CoA, as occurs in normal cells. We found increased glycolysis in breast tumors vs. normal breast, in Luminal B vs. Luminal A tumors, in HER2 vs. Luminal A tumors, in TNBC vs. luminal A tumors, and in metastasis vs. primary breast tumors, corroborating previous reports that glycolysis is related to aggressiveness in breast tumors [31]. Although it has recently been reported that the main upregulated pathway in micrometastasis is OXPHOS [32], we found both OXPHOS and glycolysis to be a hallmark in metastasis. The availability of nutrients in metastatic niche is different than in primary tumors, and metabolism is not the same in all metastatic niches. After POU1F1 overexpression, we found a significant increase in the glycolysis signature. Microarray data were confirmed by qPCR, showing increased mRNA expression of multiple enzymes belonging to the glycolysis pathway, as well as elevated mRNA levels of glucose GLUT1 and lactate MCT4 transporters in MCF7 cells after POU1F1 overexpression. MCT4 lactate transporter is a critical determinant of disease prognosis and glycolytic metabolism. Targeting MCT4 suppresses tumor growth and yields vulnerability to metabolic stress [33]. In addition, high lactate levels are present in CM of MCF7-POU1F1 cells, demonstrating increased aerobic glycolysis and lactate secretion. Functionally, metabolic analyses in either MCF7 cells with POU1F1 overexpression or MDA-MB-231 cells after POU1F1 knockout indicate that POU1F1 reprograms cancer cell metabolism toward a glycolytic pattern. Importantly, primary cultures of human breast tumors with low and high POU1F1 expression showed similar metabolic results to those obtained in cell lines after POU1F1 knockout and overexpression.

The LDHA enzyme is a key component of the glycolysis pathway. However, only a few factors that transcriptionally regulate LDHA are currently known. Among them, the c-Myc oncogenic transcription factor transactivates *LDHA* gene and stimulates aerobic glycolysis [34], and the HIF-1α transcription factor upregulates the rate-limiting enzyme for glycolysis (GLUT1 transporter) and transcriptionally regulates LDHA [35, 36]. Our study showed that *LDHA* mRNA is highly expressed in tumors with respect to normal breast and we found a significant correlation between *POU1F1* and *LDHA* mRNA expression. In fact, two POU1F1 binding sites were identified in the promoter region of the *LDHA* gene, and luciferase reporter assays demonstrated that POU1F1 transcriptionally regulates the *LDHA* gene. Interestingly, we found that the histone mark H3K27ac, which is related to active enhancer regions, is increased in TNBC as compared with luminal breast cancer cell lines. This suggests an increased transcriptional activity of POU1F1 and consequently increased expression of LDHA in highly aggressive breast cancer cell lines [37]. At functional level, the upregulation of LDHA by POU1F1 interferes with mitochondrial respiration, turning cancer cell metabolism into aerobic glycolysis instead of OXPHOS. Furthermore, pharmacological and genetic LDHA blockade reduces cell migration and invasion and, interestingly, decreases breast cancer cell proliferation under hypoxic environment. Because POU1F1 does not modify HIF-1α expression (data not shown), our data suggest that POU1F1 regulates LDHA in hypoxic cells independently of HIF-1α. Our results are in line with those previously obtained by Leder’s group, who demonstrated that LDHA knockdown resulted in a compromised ability of tumor cells to proliferate under hypoxia [7]. In vivo, LDHA knockdown in POU1F1-overexpressing tumors reduces cell proliferation and tumor growth, and, importantly, decreases [^18^F]FDG uptake. In fact, [^18^F]FDG PET/CT imaging is widely used in clinical practice for staging and follow-up after cancer treatment.

In addition to its role in breast tumors, POU1F1-mediated glycolytic activity could also have important physiological and pathological consequences. POU1F1 plays a key role in cell differentiation during anterior pituitary gland organogenesis in mammals [38], and the POU family of transcription factors (POU1F1, Oct, and Unc) are critical in key biological processes, such as cell proliferation, determination of cell lineage fate and regulation of cell migration, survival, and terminal differentiation [39]. Furthermore, as commented above, increased LDHA-mediated glycolysis is related to invasion and proliferation of pituitary adenomas [17]. Given that POU1F1 is mainly expressed in the pituitary gland, we could speculate that POU1F1-LDHA-mediated glycolysis plays a role in pituitary cell differentiation and proliferation.

TME consisting of tumor cells and host stromal cells has emerged as an important player in tumor progression. Among stromal cells, macrophages and fibroblasts are the major components of tumor mass. Recently, we have demonstrated that POU1F1 overexpression induces both recruitment of monocytes-macrophages to breast tumor area and polarization of macrophages into TAM [40]. In the present study, we evaluated the role of POU1F1 in fibroblast activation. CAFs are central elements of the TME, interacting with tumor cells and other stromal cells [41]. Although CAFs may have tumor suppressive properties, they mainly have tumor promoting capacities. In fact, high CAF presence in TME is related to cancer prognosis and response to therapy [42]. Our study demonstrates a clear relationship between POU1F1 and α-SMA in breast tumors as well as the clinical prognostic value of both *POU1F1* and *ACTA2* mRNA expression. In addition, POU1F1-derived lactate induces NAF into CAF activation, which is partially reverted by either POU1F1 knockdown or LDHA blockade. In some tumors, lactate has recently been reported to epigenetically reprogram mesenchymal stem cells to differentiate into CAFs, thus leading to tumor progression [15]. In addition, lactate binds to the GPR81 lactate receptor, which is highly expressed in cancer cells, including breast cancer cells [43, 44]. GPR81 is also expressed in stromal cells, such as antigen presenting dendritic cells, suggesting that tumoral lactate prevents dendritic stromal cells from presenting cancer cell-specific antigens to other immune cells, which may be of importance to cancer cell immune evasion [45]. The present study evaluated neither epigenetic reprogramming of NAFs through POU1F1-induced lactate nor GPR81 lactate receptors in NAFs, but we cannot exclude either possibility in lactate-mediated NAF to CAF activation.

Altogether, our data suggest that LDHA and POU1F1 could be important therapeutic targets in cancer. Interestingly, Sherman et al. [46] have demonstrated that pancreatic stellate cells, the predominant fibroblastic cell type in the TME of pancreas tumors, are reconverted to quiescent state by stromal remodeling after vitamin D treatment, indicating a molecular strategy to transcriptional reprogramming of tumor stroma. POU1F1 is transcriptionally repressed by vitamin D and its analogs [47, 48]. This led us to hypothesize that vitamin D treatment alone and/or in combination with LDHA inhibitors [49] might be a therapeutic strategy in breast cancer, acting on both cancer and stromal cells to potentiate the chemotherapeutic response in breast tumors. The effect of both treatments in glycolytic breast tumors should be further elucidated.

## Material and methods

### Cell culture, reagents, and CM

The human breast adenocarcinoma MCF7 and MDA-MB-231 cell lines were obtained from the European Collection of Cell Culture (ECACC; Porton Down, UK). MCF7 Tet-Off cells were purchased from Clontech-Takara (Kusatsu, Japan). All cells were negative and periodically tested for mycoplasm. Cell lines were cultured in DMEM supplemented with 10% FBS (Gibco, Paisley, UK) and grown in an air-CO_2_ (95:5) atmosphere at 37 °C. Hypoxic experiments were carried out at 1% O_2_. The LDHA inhibitor used at 10 μM in all experiments was purchased from Tocris (GSK 2837808 A, Minneapolis, USA). Lactate (Sigma-Aldrich, San Luis, USA) was used at 20 mM. CM was obtained after seeding the cells at 2 × 10^5^ cs/cm^2^ in DMEM with 10% FBS and cultured overnight. Afterward, cells were washed in PBS and cultured again in DMEM/F12 without FBS for 24 h. Medium was centrifuged for 5 min at 300 g and the supernatant was collected and used immediately or stored at −80 °C.

Details of additional methodology can be found in Supplementary Data.

## Supplementary information

Supplemental Material
