# Supplementary material for: POU1F1 transcription factor induces metabolic reprogramming and breast cancer progression via LDHA regulation
Source: Oncogene. 2021 Mar 13;40(15):2725–40. doi: 10.1038/s41388-021-01740-6 (PMC8049871; doi:10.1038/s41388-021-01740-6)
Supplement: Supplementary file 1 — Supplemental Material [file 41388_2021_1740_MOESM1_ESM.doc]

**SUPPLEMENTARY DATA**

**POU1F1 transcription factor induces metabolic reprogramming and breast cancer progression via LDHA regulation**

Anxo Martínez-Ordoñez, Samuel Seoane, Leandro Avila, Noemi Eiro, Manuel Macia, Efigenia Arias, Fabio Pereira, Tomas García-Caballero, Noemi Gómez-Lado, Pablo Aguiar, Francisco Vizoso, Roman Perez-Fernandez

**SUPPLEMENTARY MATERIAL AND METHODS**

***Cell culture****.* Stock cultures were grown in 90-mm Petri dishes in DMEM supplemented with 10% FBS, 100 U/ml penicillin, 100 μg/ml streptomycin and 2mM L-glutamine in an air-CO_2_ (95:5) atmosphere at 37ºC. Confluent cells were washed twice with phosphate-buffered saline and harvested by a brief incubation with trypsin-EDTA solution (Sigma Aldrich, Madrid, Spain) in PBS.

***Primary tumor cultures, normal mammary fibroblasts (NMF), normal-associated fibroblasts (NAFs), and cancer-associated fibroblasts (CAFs).*** Primary cultures of human breast tumors were obtained at Fundación Hospital de Jove (Gijón, Spain) according to institutional guidelines. The study adhered to National regulations and was approved by the institution's Ethics and Investigation Committee. After tumor resection, an experienced pathologist examined and obtained a representative piece of tumor tissue. Tumors were mechanically disaggregated by mincing with scalpel and scissors to 1-2 mm^3^ in a 6-well plate. Tissue was digested with 1.25 mg/ml of collagenase A in complete-DMEM/F12 medium for 48 hours in an air-CO_2_ (95:5) atmosphere at 37ºC. NAFs, CAFs, and NMFs were isolated as previously described (1). Briefly, CAFs were isolated from tumor tissue, NAFs from tissue corresponding to surgical margins free of tumor (at least 2 cm away from the tumor), and NMFs were obtained from healthy woman after breast mastectomy. Culture purity of NAFs, CAFs and NMFs was confirmed by flow-cytometry using the anti-CD90 antibody (clone AS02, Dianova, Hamburg, Germany). Primary cultures were grown in DMEM/F12 medium, supplemented with 10% FBS, 100 U/ml penicillin, and 100 μg/ml streptomycin, 5 ng/ml epidermal growth factor (EGF), 1 μg/ml hydrocortisone, and 5 μg/ml insulin.

***Plasmids, transfections, and luciferase reporter assay.*** For transient POU1F1 overexpression, the pDream2.1/MCS plasmid (GenScript, Piscataway, USA) containing an ORF clone of the human POU1F1 gene (pDream-POU1F1) and the pDream empty vector (as control) were used. Transient POU1F1 blockade was performed using a pool of 3 target-specific lentiviral vector plasmids each encoding 19-25 nt (plus hairpin) shRNAs to knock-down POU1F1 gene expression (Santacruz Biotechnology, Heidelberg, Germany). A pool of 3 scrambled shRNA sequence were used as control. Transfections were performed with X-tremeGENE (Roche, Basel, Switzerland) according to the manufacturer’s instructions. Stable transfection of the MCF7 Tet-Off cells (Clontech-Takara, Saint-Germain-en-Laye, France) with the pTRE2 control vector and the pTRE2-POU1F1 overexpression vector was performed as previously described (2). POU1F1 knock-out in the MDA-MB-231 cells was carried out using the pLentiCRISPRv2 plasmid with the guide sequence for POU1F1: CTTACCTGCCATCACTCCAT (GenScript). Control cells were transfected with the pLentiCRISPRv2 guide control (Addgene, Watertown, USA). After 96 h a serial dilution was performed to obtain single cells. Survival clones were selected with puromycin dihydrochloride (2 µg/ml). Knock-down of the LDHA gene was carried out by transfecting the pLKO-shLDHA vector (Sigma) in the MCF7 Tet-Off cells with POU1F1 overexpression. The empty vector was used as control. The shRNA targeting sequences were the following: LDHA shRNA1 (TRCN0000158441), and LDHA shRNA2 (TRCN0000164922). After 96 h, knock-down cells were selected with puromycin dihydrochloride (2 µg/ml). For LDHA luciferase reporter assays,  HEK 293 cells were transfected in 6-well plates containing 6 μl of jetPRIME Polyplus transfection reagent (PolyPlusTransfection, Illkirch, France), 2 μg of pDream (empty vector) or pDream-POU1F1 overexpression vector, 1 μg of each reporter plasmid: pRP-LDHA_promoter_-WT (wild promoter, from -101 to +315 bp with respect to transcription start site), pRP-LDHA_promoter_-DEL1 (-101 to +315 bp with a deletion of the POU1F1 binding site at position -76 to -62 bp), and pRP-LDHA_promoter_-DEL2 (-101 to +315 bp with a deletion of the POU1F1 binding site at position +276 to +290 bp) (VectorBuilder, Chicago, USA), and 50 ng of pRL-TK-Renilla (as transfection control) for 48 h. The cells were lysed in buffer (500 μl lysis buffer, Promega Corporation, Madison, USA) and luciferase was measured in a Mithras LB 940 apparatus (Berthold Technologies, Bad Wildbad, Germany).

***RNA isolation and quantitative PCR (qPCR).*** Total RNA was isolated with TRIzol (Invitrogen, Carlsbad, USA). Human breast tumor samples (n=21) were provided by the BioBank from the Complejo Hospitalario Universitario de Santiago (CHUS), integrated in the Spanish National Biobanks Network and were processed following standard operating procedures. Approval by the Ethical and Scientific Committees was obtained. cDNA was synthesized with M-MLV-RT (Invitrogen), and reactions of quantitative real time PCR were done using SYBR Green PCR Master Mix (Thermo Scientific, Waltham, USA) on StepOnePlus Real time PCR System (Applied Biosystems). The samples were denatured at 95°C for 5 sec, annealed at 55°C for 10 sec and extended at 72°C for 40 sec, for a total of 40 cycles. The samples were quantified using the Sequence Detection Software 1.4 (Applied Biosystems), with 18S as normalization control. The oligonucleotide sequences used in real-time PCR were as follows:

| **gene** | **forward** | **reverse** |
| --- | --- | --- |
| *POU1F1* | attcttgacgcctctgcaact | ccataggttgatggctggtt |
| *CXCL12* | cgattcttcgaaagccatgt | ctttagcttcgggtcaatgc |
| *VEGFA* | cgcaagaaatcccggtataa | aaatgctttctccgctctga |
| *ACTA2* | agaacatggcatcatcacca | tacatggctgggacattgaa |
| *VIM* | gagaactttgccgttgaagc | tccagcagcttcctgtaggt |
| *FAP* | tacccaaaggctggagctaa | acaggaccgaaacattctgg |
| *18S* | gtaacccgttgaaccccatt | ccatccaatcgctagtagcg |
| *GPI* | tattgtgttcaccaagctcacacc | tggtagaagcgtcgtgagaggtc |
| *PFKL* | ggagaagctgcgcgaggtttac | attgtgccagcatcttcagcatgag |
| *ALDOA* | aggccatgcttgcactcagaagt | agggcccagggcttcagcagg |
| *GAPDH* | ttccgtgtccccactgccaacgt | caaaggtggaggagtgggtgtcgc |
| *PGK1* | atgtcgctttctaacaagctga | gcggaggttctccagca |
| *PGAM1* | ggaaacgtgtactgattgcagccc | ttccatggctttgcgcaccgtct |
| *ENO1* | gacttggctggcaactctg | ggtcatcgggagacttgaa |
| *ENO2* | tcatggtgagtcatcgctcaggag | atgtccggcaaagcgagcttcatc |
| *PKM2* | gcccgtgaggcagaggctgc | tggtgaggacgattatggccc |
| *GLUT1* | catcccatggttcatcgtggctgaact | gaagtaggtgaagatgaagaacagaac |
| *GLUT4* | ttttgagattggccctggccccat | ctcaggtactcttaagaaggtgaag |
| *LDHA* | actgcaaactccaagctggt | ctggatttgaaacaataagcaa |
| *MCT4* | gcacccacaagttctccagt | caaaatcagggaggaggtga |
| *TIGAR* | agtgttccttaccagccact | agcgagtttcagtcagtcca |

***Cell lysis, Western blot, immunofluorescence (IF) and immunohistochemistry (IHC)*.** For protein analysis, cells were lysed in buffer (20 mM Tris-HCl pH=7, 140 nM NaCl, 50 mM EDTA, 1% Triton-X, 10% glycerol, with phosphatase and protease inhibitors). Tumor tissues from mice were homogenized using a TissueLyser II (Qiagen, Hilden, Germany). For Western blotting, 15 µg of total protein was subjected to SDS-PAGE electrophoresis and proteins transferred to Immobilon-FL membranes (Merck Millipore, Madrid, Spain), blocked with milk and incubated overnight at 4 °C with primary antibodies. The anti-mouse Dylight™ 680-conjugated and anti-rabbit Dylight™ 800-conjugated (Thermo Fisher Scientific) were used as secondary antibodies. Quantification was performed using Image Studio Lite 5.2 (Bad Homburg, Germany). The following antibodies were used:

| **Antibody** | **Application** | **Source** |
| --- | --- | --- |
| POU1F1 | WB  IHC  IF | Aviva (ARP32318_P050)  Abmart  Santa Cruz (X-7) |
| LDHA | WB, IF | Santa Cruz (sc-137243) |
| α-SMA | WB  IHC | Abcam (ab5694)  Dako-Agilent (clone 1A4, IR611) |
| Ki-67 | IHC | Dako-Agilent (clone MIB-1,GA626) |
| β-actin | WB | Santa Cruz (sc-47778) |

For IF 15 x 10^3^ cells were seeded in p24 well plates containing coverslips coated with Poly-L-Lysine (Sigma), fixed in cold methanol overnight, blocked and permeabilized. Cells were incubated with the primary and the corresponding Alexa-488/555 secondary antibodies (Thermo Fisher Scientific). DAPI (4′,6-diamidino-2-phenylindole) was used for nuclear staining. Dragonfly confocal spinning-disk on a Nikon Eclipse Ti-E equipped with an Andor Zyla 4.2 PLUS sCMOS digital camera was used to visualize the IF. For IHC, mice tumor xenografts and human breast tumors tissues were immersion fixed in 10% neutral buffered formalin for 24 h and embedded in paraffin routinely. Sections 4 µm-thick were mounted on FLEX IHC microscope slides (Agilent, Carpinteria, USA). After deparaffination and epitope retrieval (for 20 min at 97ºC in EnVision FLEX target retrieval solution at low pH for Ki67 and high pH for Pit1 and α-SMA), immunohistochemistry was automatically performed using an AutostainerLink 48 immunostainer (Dako-Agilent). Briefly, the slides were incubated at room temperature: 1) POU1F1 mouse monoclonal antibody generated by Abmart Inc. (Shanghai, China) at 1:100 for 30 min, α−SMA ready to use monoclonal antibody (Dako-Agilent) for 20 min, and Ki67 ready to use monoclonal antibody (MIB1, Dako-Agilent), for 30 min + Envision FLEX+ Mouse Linker (Dako-Agilent); 2) EnVision FLEX/HRP (dextran polymer conjugated with horseradish peroxidase and affinity-isolated goat anti-mouse immunoglobulins) for 20 min; 3) substrate working solution (mix) (3,3’ diaminobenzidine tetrahydrochloride chromogen solution) for 10 min; and 4) EnVision FLEX hematoxylin for 9 min. Immunostaining results were evaluated by an expert pathologist.

***Proliferation, migration assay and organotypic cultures*.** For cell proliferation, 3 x 10^4^ cells were seeded in triplicate in 24-well plate. After treatments for 72 h in normoxic or hypoxic conditions, cells were trypsinized and counted with a Neubauer hemocytometer (Celeromics, Cambridge, UK). MCF7, MCF7-POU1F1 (treated with DMSO or transfected with the pLKO-control vector), MCF7-POU1F1 treated with LDHAi (10 µM), and MCF7-POU1F1 cells transfected with the pLKO-shLDHA vector were assayed for their ability to migrate through a polyethylene terephthalate membrane in a 24- well using the Corning BioCoat™ Insert System (Corning, New York, USA). A total of 2.5 ×10^4^ cells per well were seeded into the inner chamber in serum-free DMEM. DMEM was placed in the outer chamber containing 20% FBS. After 16 h, cells that migrated through the pores onto the bottom of the insert were fixed in 100% cold methanol and stained with crystal violet (Sigma). The total number of migrating cells was determined by counting the cells on the lower surface of the insert using a light microscope. Organotypic cultures were performed as described previously (3). Organotypic gels were harvested, fixed in 10% neutral buffered formalin, bisected, and embedded in paraffin. H&E stained sections were analyzed with a BX43 Olympus light microscope. Quantification of invading cells was performed as described previously (3) using ImageJ software.

***Proton nuclear magnetic resonance (^1^H-NMR)*.** MCF7 or MCF7-POU1F1 cells were seeded at 1.5 x 10^6^ in p60 plates in DMEM without FBS and after 24 h medium was centrifuged and supernatant collected and stored at -80 ºC until analysis. The exometabolome analysis of CM-MCF7 and CM-MCF7-POU1F1 was performed by ^1^H-NMR as previously described (4). Identification of metabolites was done using the Chenomx NMR Suite 8.1 (Chenomx Inc., Alberta, Canada) and relative concentrations (in µM) were determined using the 500 MHz library from Chenomx NMR Suite 8.1, which compares the integral of a known reference signal (TSP) with signals derived from a library of compounds containing chemical shifts and peak multiplicities.

***Glycolytic Rate Assay*.** Glycolytic activity of breast cancer cells (25 x 10^3^ cells for primary cultures and 15 x 10^3^ cells for MCF7, MCF7-POU1F1, MDA-MB-231, MDA-MB-231sgPOU1F1 and MCF7-POU1F1-shLDHA) was determined using an XFp Extracellular Flux Analyzer (Seahorse Bioscience, Billerica, USA). Cells were seeded in triplicate into XFp microplates. For LDHA inhibitor treatment, breast cancer cells were seeded and after 4 h culture medium was replaced by medium plus treatment or vehicle. One day later, culture medium was changed to XF basal medium with 7.4 pH containing 10 mM glucose, 1 mM pyruvate and 2 mM Glutamine. The plate was incubated at 37°C in a non-CO_2_ incubator for 60 min before extracellular flux analysis. After four baseline measurements, glycolytic parameters were calculated using 0.5 μM of Rotenone/Antimycin A, a mix of Complex-I,III-dependent respiration inhibitors and 5 μM of 2-DG (2-deoxy-D-glucose) as inhibitor of glycolysis (hexokinase inhibitor) to confirm pathway specifity. Values were normalized to cell number (1000 cs) in all conditions.

***Animal studies and Positron Emission Tomography/Computer tomography (PET/CT) imaging.*** All animal studies were approved by the University of Santiago de Compostela Ethics Committee for Animal Experiments. Female mice (age matched, 8 weeks) with immunodeficiency (BALB/cAnNRj-Foxn1^nu/nu^, referred to as BALB/c-nu, (Janvier Labs, Le Genest St Isle, France) were used for xenografting studies. Orthotopic primary tumors were generated in mice by inoculation into the mammary fat pad of 5 × 10^6^ MCF7 cells stably transfected with the pTRE2 control vector (n=7, controls), the pTRE2-POU1F1-overexpressing vector plus the pLKO vector (n=7), and the pTRE2-POU1F1–overexpressing vector plus the pLKO-shLDHA vector (n=7) in 0.2 ml of DMEM without FBS and Matrigel (50:50, BD Biosciences). Tumor growth was monitored externally using a digital calliper every three days, until day 15. Tumor volume was calculated using the following formula: length × width^2^/2.

PET/CT studies using ^18^F-Fluoro-2-deoxy-2-D-Glucose ([^18^F]FDG) were performed 16 days after tumor cell injection in 12 mice (four mice per group, 16 days post-induction, 17.25 ± 1.03 g) to study tumor metabolism between different groups. All animals were fasted for 12 hours before the radiotracer injection. PET/CT images were acquired using an Albira PET/CT Preclinical Imaging System (Bruker Biospin, Woodbridge, USA). After an overnight fast, 3.97 ± 0.71 MBq of [^18^F] Fluoro-2-deoxy-2-D-Glucose ([^18^F] FDG) was injected in the tail vein of each animal. Then, mice remained at rest during 40 min with access to water. Next, mice were placed in a gas chamber containing 3% isoflurane in oxygen until they were unconscious. Standardized Uptake Value (SUV_max_) was calculated as the maximum [^18^F]FDG uptake value normalized by the injected activity and animal body weight as follows: SUV_max_ = (maximum tracer uptake in tumour * weight) / injected activity. The injected [^18^F]FDG activity was estimated by subtracting the extravasated activity in tail. Finally, PET/CT static acquisitions were performed after a standardized uptake time of 45.58 ± 1.51 min, consisting of 10 min PET scan followed by 20 min CT scan. No contrast agent was administered to carry out the CT scan. PET/CT images were reconstructed at the end of the acquisition. All images were analyzed using AMIDE software. Fused [^18^F]FDG PET/CT images were used to define the tumor region in every mouse. Then, quantitative analysis was carried out by using circularly delineated Region of Interest (ROI) on CT images. The ellipsoidal ROIs dimensions outlined the corresponding tumor with a volume of 9.82 ± 3.42 mm^3^ (3.27 ± 0.67 mm in x-axis, 2.80 ± 0.66 mm in y-axis and 2.05 ± 0.19 mm in z-axis). Subsequently, the ROIs were transferred to PET images to calculate the maximum [^18^F]FDG uptake value.

***Gene expression and bioinformatic analysis.*** Microarray assay of mRNA was performed using an Affymetrix Human Gene 1.0 ST Array (GEO database access no. GSE64101). Gene expression (.gct) and phenotype labels (.cls) were used as input for Gene set enrichment analysis generated according to GSEA v2.0.14 software instructions (http://www.broadinstitute.org/gsea/index.jsp). GSEA was run under 1000 gene‐set permutations using the gene‐ranking metric t-test with H MSigDb collection. Gene expression (GSE23988, GSE109169, GSE45827 and GSE103357) data were obtained from GEO (<https://www.ncbi.nlm.nih.gov/geo/>). Breast cancer samples (GEO: GSE22820 and Biobank) were segregated into two subgroups based on POU1F1 expression: POU1F1high (higher than 75th percentile) and POU1F1low (with levels below 25th percentile). The bigwig files of ChIPseq data, H3K27ac (accession number GSE69112) from human breast cancer cell were downloaded from ENCODE (https://www.encodeproject.org/search/?type=experiment). The binding signals on the specific genomic loci were visualized using IGV_2.3.53 program downloaded from <http://www.broadinstitute.org/software/igv/node/250>. The KM Plotter Online (http://kmplot.com) was used to evaluate how patient clinical outcome is related to POU1F1/LDHA, POU1F1/ACTA2 and LDHA/ACTA2 mRNA expression (5). PROGgeneV2, Pan Cancer Prognostics software was used to evaluate overall survival based on POU1F1 and LDHA expression (6). Transcription factor motif analysis of LDHA promoter was performed using JASPAR (<http://jaspar.genereg.net/>).

***Statistical Analysis.*** Statistical analysis was performed using GraphPad Prism 7 software (San Diego, USA). Data was presented as the mean ± SEM, unless specified. Significant differences between groups were determined using a Student’s t test when the data were normally distributed according to the D’Agostino test. Otherwise, the Mann-Whitney test was used. For multiple comparisons, we applied a false discovery rate (FDR) approach. An FDR adjusted p-value (q-value) < 0.05 was considered significant. All experiments were performed at least three times, unless otherwise noted. Gene expression correlation analyses were performed using Pearson’s correlation coefficients when the data met a Gaussian distribution. Otherwise, a Spearman’s correlation was used. The significance level for statistical testing was set at *P* < 0.05 (**P*<0.05, ***P*<0.01, *P****<0.001).

**Supplementary References**

1. González L, Eiro N, Fernandez-Garcia B, González LO, Dominguez F, Vizoso FJ. Gene expression profile of normal and cancer-associated fibroblasts according to intratumoral inflammatory cells phenotype from breast cancer tissue. *Mol Carcinog*. 2016;55(11):1489-1502.
2. Ben-Batalla I, et al. Deregulation of the Pit-1 transcription factor in human breast cancer cells promotes tumor growth and metastasis. *J Clin Invest* 2010; **120**:4289-4302.
3. Ranftl RE, Calvo F. Analysis of Breast Cancer Cell Invasion Using an Organotypic Culture System. *Methods Mol. Biol* 2017; **1612**:199-212.
4. Duarte TM, Carinhas N, Silva AC, Alves PM, Teixeira AP. ^1^H-NMR Protocol for Exometabolome Analysis of Cultured Mammalian Cells. *Methods Mol Biol* 2014; **1104**:237-247.
5. Gyorffy B, et al. An online survival analysis tool to rapidly assess the effect of 22,277 genes on breast cancer prognosis using microarray data of 1809 patients. *Breast Cancer Res Treat* 2010; **123**:725-731.
6. Goswami CP, Nakshatri H. PROGgeneV2: enhancements on the existing database. *BMC Cancer* 2014; **14**:970.

Supplementary Fig. S1

**Supplementary Fig. S1.** Dataset enrichment graphs related with metabolic and proliferation process categories from Hallmark collection and GSEA plot of enrichment in glycolysis geneset. Gene expression data obtained from GSE45827. (A) Luminal B breast tumors (n=30) vs Luminal A breast tumors (n=29); (B) HER2 breast tumors (n=30) vs Luminal A breast tumors (n=29). FDR: false discovery rate; NES: normalized enrichment score.

Supplementary Fig. S2

**A**

**B**

**Supplementary Fig. S2.** (A) Representative ^1^H-NMR spectral peaks of glucose, pyruvate, and lactate in CM-MCF7. (B) Representative ^1^H-NMR spectral peaks of glucose, pyruvate, and lactate in CM-MCF7-POU1F1.

Supplementary Fig. S3

**Supplementary Fig. S3.** Western blot of three MDAsgPOU1F1 clones showing POU1F1 and β-actin expression.

Supplementary Fig. S4

**Supplementary Fig. S4.** (A) Quantitative Western blot of POU1F1, LDHA, and β-actin in MCF-7 cells and MCF7-POU1F1 cells. (B) WB of POU1F1, LDHA and β-actin in control MDA-MB-231 cells and after POU1F1 knock-out (three clones, MDAsgPOU1F1 cells). (C) WB of LDHA, POU1F1 and β-actin in MCF7, MCF7-POU1F1, and in two clones of MCF7-POU1F1 with transient knock-down of LDHA (MCF7-POU1F1-shLDHA2 and shLDHA3). n=2

Supplementary Fig. S5

5


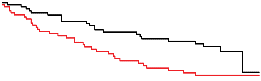


1.0

0.8

0.6

0.4

0.2

0.0

0

1

2

3

4

OS probability

High POU1F1-LDHA

Low POU1F1-LDHA

HR=6.38 (1.42-28.67)

P=0.015

Days (x10^3^)

GSE3494

N=236

1.0

0.8

0.6

0.4

0.2

0.0

0

1

2

3

OS probability

High POU1F1-LDHA

Low POU1F1-LDHA

HR=8.08 (1.99-32.9)

P=0.003

Days (x10^3^)


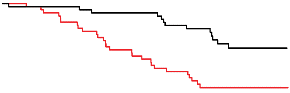


GSE1456

N=40


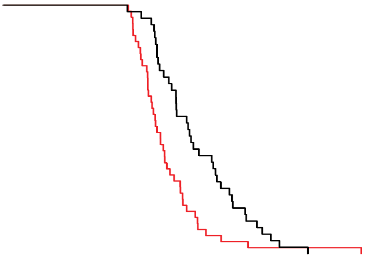


1.0

0.8

0.6

0.4

0.2

0.0

0

1

2

3

4

5

High POU1F1-LDHA

Low POU1F1-LDHA

HR=2.37

(1.39-4.03)

P=0.001

Days (x10^3^)

GSE21653

N=79

OS probability

5


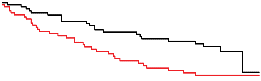


1.0

0.8

0.6

0.4

0.2

0.0

0

1

2

3

4

OS probability

High POU1F1-LDHA

Low POU1F1-LDHA

HR=6.38 (1.42-28.67)

P=0.015

Days (x10^3^)

GSE3494

N=236

1.0

0.8

0.6

0.4

0.2

0.0

0

1

2

3

OS probability

High POU1F1-LDHA

Low POU1F1-LDHA

HR=8.08 (1.99-32.9)

P=0.003

Days (x10^3^)


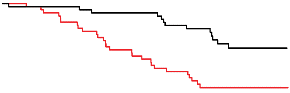


GSE1456

N=40


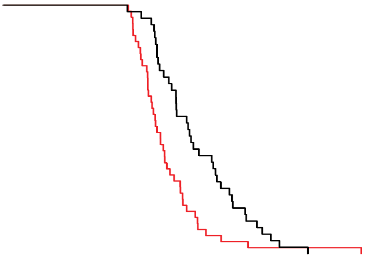


1.0

0.8

0.6

0.4

0.2

0.0

0

1

2

3

4

5

High POU1F1-LDHA

Low POU1F1-LDHA

HR=2.37

(1.39-4.03)

P=0.001

Days (x10^3^)

GSE21653

N=79

OS probability

**Supplementary Fig. S5**. Correlation between POU1F1 and LDHA mRNA expression and overall survival (OS) in human breast tumors. Analysis was done using the ProgGeneV2 online tool.

Supplementary Fig. S6

**Supplementary Fig. S6.** (A-C) Normal-associated fibroblasts (NAFs) and cancer-associated fibroblasts (CAFs) were characterized in three primary cultures of breast cancer patients (see Suppl. Table 4). Vimentin (VIM), fibroblast activation protein alpha (FAP), vascular endothelial growth factor A (VEGF) and C-X-C motif chemokine 12 (CXCL12) mRNA were evaluated by qPCR in NAFs and CAFs from the 035 patient (A), the 823 patient (B), and the 2920 patient (C). Data are expressed as mean ± SEM. **P*<0.05, ***P*<0.01, and ****P*<0.001.

Supplementary Fig. S7

**Supplementary Fig. S7**. (A) Quantitative WB of α-SMA and β-actin in normal mammary fibroblasts (NMF) after treatment for 24 h with conditioned medium (CM) from NMF (CM-NMF), MCF7 (CM-MCF7) and MCF-7-POU1F1 (CM-MCF-7-POU1F1) cells. (B) WB of α-SMA and β-actin in NMF after treatment for 24 h with CM-NMF, CM-MDAMB231 (CM-MDAsgC) and CM-MDAMB231 cells after POU1F1 knock-out (CM-MDAsgPOU1F1). n=2.

Supplementary Fig. S8

**Supplementary Fig. S8**. (A) WB of α-SMA and β-actin in NMF and NAF823 after administration of 0, 5, 10, and 20 mM lactate for 24 h. (B) Conditioned medium (CM) from 24 h culture of control MCF7-POU1F1 cells, and after pharmacological (MCF7-POU1F1-LDHAi) or genetic (MCF7-POU1F1-shLDHA2) LDHA inhibition was added to NAF823 for 24 h and α-SMA and β-actin protein levels were evaluated by WB. (C) Normal fibroblasts (NMF) were treated as in (B). (D) WB of α-SMA and β-actin in NAF823 after administration of CM-NAF823, CM-MCF7-POU1F1, and CM-MCF7-POU1F1+LDHAi for 24 h. (E) WB of α-SMA and β-actin in NAF823 after administration of CM-NAF823, CM-MCF7-POU1F1, and CM-MCF7-POU1F1-shLDHA2 for 24 h. (F) WB of α-SMA and β-actin in NMF after administration of CM-NMF, CM-MCF7-POU1F1, and CM-MCF7-POU1F1-LDHAi for 24 h. (G) WB of α-SMA and β-actin in NMF after administration of CM-NMF, CM-MCF7-POU1F1, and CM-MCF7-POU1F1-shLDHA for 24 h.

**Supplementary Table S1**. Metabolite concentration (µM) (sugars –blue-, organic acids –orange-, amino acids –green-, and others -red-) in conditioned medium of control (M1-M4) and POU1F1-overexpressed (M5-M8) MCF7 cells analysed by ^1^H-NMR.

**Supplementary Table S2.** Basal characteristics of 21 patients with breast cancer used for mRNA analysis**.**

**Supplementary Table S3.** Basal characteristics of 7 patients with breast cancer used for primary cultures and protein analysis.

**Supplementary Table S4**. Clinical data of three human breast tumors (035, 823, and 2920) used for NAF and CAF isolation and experimental use.
